# Numerical simulation of CO generation and migration patterns in goaf based on coupled multi-physics fields

**DOI:** 10.1038/s41598-026-55032-8

**Published:** 2026-05-23

**Authors:** Mengxuan Ren, Yongli Liu, Bingkun Duan, Xinxu Li, Haitao Wang

**Affiliations:** 1 School of Safety Engineering, Heilongjiang University of Science and Technology, Harbin, 150022 China; 2 School of Mining Engineering, Heilongjiang University of Science and Technology, Harbin, 150022 China

**Keywords:** Coal spontaneous combustion, Thermal buoyancy, Indicator gas, Multi-physics coupled simulation, Programmed heating experiment, Energy science and technology, Engineering, Environmental sciences

## Abstract

To explore early prediction methods for goaf spontaneous combustion, a numerical simulation was conducted to investigate the generation and migration laws of indicator gases in the goaf. A multi-physics coupling model integrating flow field, temperature field and concentration field was adopted to systematically analyze the spatio-temporal evolution of the temperature field and indicator gases. Programmed heating experiments revealed that CO exhibits a good correlation with temperature at low stages. Accordingly, CO was determined as the indicator gas in the numerical simulation, and the oxygen consumption rate, CO generation rate and heat release intensity were obtained. The multi-physics coupling results demonstrate that thermal buoyancy is the dominant driving force controlling the vertical migration and spatial distribution of CO in the goaf. Under the combined effect of air leakage and thermal buoyancy, CO accumulates in the upper, deep and return side regions of the goaf, which should be prioritized for monitoring. The findings provide important theoretical and engineering support for the prevention and control of goaf spontaneous combustion.

## Introduction

Coal remains one of the world’s most important primary energy sources, and despite the accelerating global transition to cleaner energy, it is expected to maintain a significant share in the primary energy consumption structure for a considerable period^1,2^. Spontaneous combustion of residual coal in mine goafs is a major hazard threatening safe production. The carbon monoxide (CO) released during this process not only easily triggers fires and explosions but also may cause worker poisoning, severely restricting the safe and efficient exploitation of coal resources^3–5^. Therefore, an in-depth analysis of CO generation mechanisms and migration patterns in goafs is crucial for early warning of coal spontaneous combustion, optimizing fire prevention technologies, and ensuring mine safety.

Extensive research has been conducted on gas (mainly CH₄) migration in goafs based on porous media seepage theory. Early numerical simulation studies established fundamental models for gas migration. For instance, Tang Jianli^6^ built a mathematical model using Fluent by combining overlying strata damage characteristics and porous media theory, validating it with field gas drainage data. Wang Lu et al.^7^ employed COMSOL to couple the diffusion equation, seepage equation, and Darcy’s law, clarifying gas sources and migration laws. Subsequently, multi-field coupling models emerged. Xu Zhenwei^8^ constructed a coupled convection–diffusion model for gobs with gob-side entry retaining. Li et al.^9^ found that larger working face airflow widens both the oxidation zone and methane explosion zone; Wang et al.^10^ developed a longwall goaf CFD model calibrated with field borehole data; Ding^11^ and Deng^12^ examined methane flow fields under Y-type ventilation; Ma et al.^13^ demonstrated an inverse relationship between airflow speed and methane concentration differentials under spontaneous combustion versus non-combustion conditions; Yu Yang^14^ simulated gas concentration distribution under U-type and Y-type ventilation using COMSOL. Although these studies have established a relatively complete theoretical and technical framework for CH_4_ migration, it is essential to clarify that CH_4_ originates primarily from coal desorption, whereas CO is mainly a low-temperature oxidation and spontaneous combustion product. The source term of CO is strongly coupled with the temperature field and chemical reaction field, and the core goal of CO migration research is to serve the early warning of spontaneous combustion—fundamentally different from CH₄ studies that often target explosion prevention or resource extraction. Compared with the mature research on CH_4_ migration, investigations into the migration mechanism, evolution law, and accurate prediction model of CO in goafs remain relatively weak. Recent advances have begun to address this gap: Wu et al.^15,16^ developed a multi-physics coupling model to describe indicator gas behaviour under thermal effects in longwall goafs. Wu Kuan^17^ established a gas–solid–thermal multi-field coupling model and analysed the spatial distribution of CO concentration field under different influencing factors. Nevertheless, research on CO migration that explicitly considers the coupling effect of coal oxidation heat generation and the detailed concentration accumulation characteristics in key areas such as the oxidation zone still requires further deepening.

In summary, previous studies have provided limited analysis of the coupled relationships among the gas concentration field, temperature field, and flow field in goafs, particularly regarding the migration and accumulation patterns of CO. To address this gap, this study develops a coupled multi-physics model integrating porous media flow, porous media heat transfer, and porous media dilute species transport. The model simulates the migration behaviour of indicator gases in the goaf under the influence of gravity, thermal buoyancy, and coal seam inclination. The findings offer practical value for improving monitoring layout in goafs and selecting appropriate locations for nitrogen injection for fire prevention and extinguishment. This work contributes to reducing the spontaneous combustion risk in goafs and ensuring safe coal mine production.

## Overview of the working face

The right first face in the 16th layer of the South Second Lower Mining Area of Dongrong No.2 Coal Mine (hereinafter referred to as the “the working face”) selected in this study is a typical downward-inclined mining face. Its coal seam and mining parameters are as follows: the coal seam thickness ranges from 2.20 m to 2.80 m, with an average thickness of 2.51 m; the dip length of the working face is 160 m at the initial mining stage, and after face reduction adjustment, the dip length is optimized to 152 m, and the strike length is about 305 m; the coal seam dip angle ranges from 12° to 20°, with an average dip angle of 16°.The coal seam is classified as a Category I highly spontaneous combustion-prone coal, with a minimum spontaneous combustion period of 30 days.

## Temperature-programmed oxidation (TPO) experiment for coal spontaneous combustion

To ensure the reliability and representativeness of experimental data, coal sample collection strictly complied with the relevant standards in the “GB/T482-2008 Methods for Collecting Coal Seam Samples”. Raw coal samples were collected on-site from the target working face. After collection, the coal samples were immediately sealed to avoid oxidation reaction with air that might affect experimental accuracy, and then quickly transported to the laboratory for standby use. During the experimental preparation stage, a jaw crusher was used to crush and screen the raw coal samples, and 1 kg of mixed-grain coal samples with a particle size range of 0.9 ~ 10 mm were selected as the test coal samples for this programmed heating experiment.

### Oxygen consumption rate and CO production rate

To investigate the generation characteristics of indicator gases during coal spontaneous combustion, oxidation experiments were conducted on the prepared coal samples using a temperature-programmed heating system. In each experiment, the coal sample was first loaded into a 0.0012 m^3^ reaction vessel, which was then placed inside a temperature- programmed oven. Air was continuously supplied into the reactor at a constant flow rate of 120 mL/min using an air pump to maintain a stable inlet oxygen concentration.

The experiment was conducted with automatic heating at a constant rate of 0.3 °C/min. The specific temperature- programming procedure was as follows: the temperature was first increased by 10 °C over a period of 10 min, followed by an isothermal hold for 20 min. During the isothermal stage, the outlet gas was collected and subjected to composition analysis. The above steps were then repeated cyclically until the temperature reached 170 °C.

To verify the applicability of different gases as early warning indicators, the variations of O_2_, CO, CO_2_, CH_4_, and C_2_ hydrocarbon gases were simultaneously monitored during the temperature-programmed experiments. The results showed that hydrocarbon gases such as C_2_H_2_, C_2_H_4_, and C_2_H_6_ were not obviously generated in the low-temperature oxidation stage below 70 °C, which is the focus of this study. Meanwhile, CO₂ and CH_4_ were significantly affected by the desorption of adsorbed gas in raw coal, leading to unstable background concentrations that could not reflect the dynamic evolution of the oxidation process. In contrast, the generation of CO exhibited a significant and stable trend with increasing temperature, showing a good correlation with the low-temperature oxidation of coal. Therefore, this study focuses on the generation and migration of CO as the core indicator for early warning of coal spontaneous combustion in the goaf. The calculation formulas for the oxygen consumption rate and CO generation rate are as follows:

1 $$\begin{array}{*{20}c} {r_{{{\mathrm{O}}_{2} }} T = \frac{{Q_{air} C_{{{\mathrm{O}}_{2} }}^{0} }}{V} \cdot \ln \frac{{C_{{{\mathrm{O}}_{2} }}^{1} }}{{C_{{{\mathrm{O}}_{2} }}^{2} }}} \\ \end{array}$$

2 $$\begin{array}{*{20}c} {{\mathrm{r}}_{{{\mathrm{CO}}}} {\text{T = }}\frac{{C_{{{\mathrm{CO}}}} }}{{C_{{{\mathrm{O}}_{2} }}^{1} - C_{{{\mathrm{O}}_{2} }}^{2} }}{\mathrm{r}}_{{{\mathrm{O}}_{{2}} }} \left( T \right)} \\ \end{array}$$

**Fig. 1:**
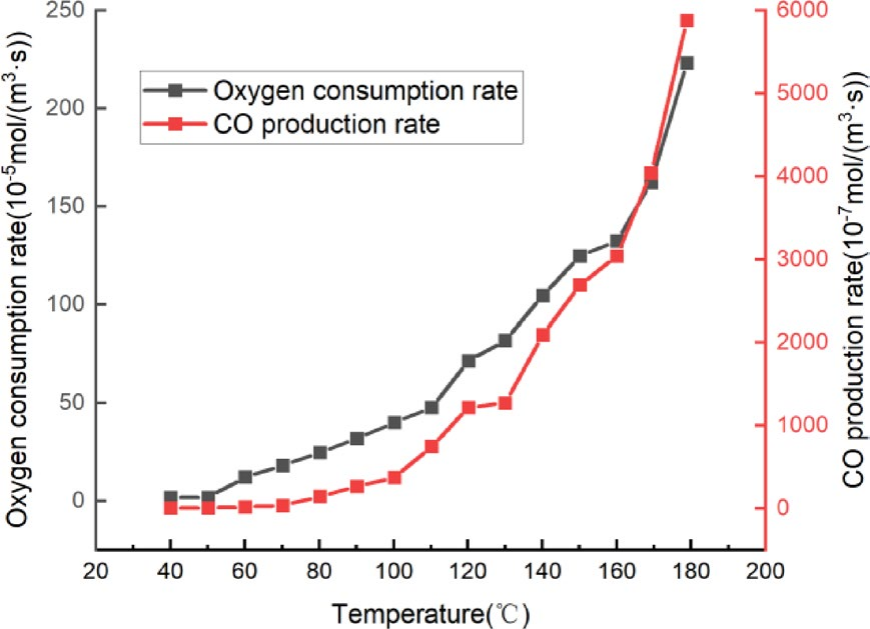
Oxygen consumption rate and CO generation rate as a function of temperature.

The experimental results of the oxygen consumption rate and CO generation rate are shown in Fig. 1.

The Arrhenius equation was employed to describe both rates, and segmented curve fitting was performed on the experimental data. The resulting segmented fitting parameters are listed in Table 1.

**Table 1:** Segmented Arrhenius parameters for oxygen consumption and CO generation.

Segment	*T*	${E}_{a}$	*A*	${R}^{2}$
${r}_{{\mathrm{O}}_{2}}$	333–383	39.7	307	0.998
	393–442	51.1	15,200	0.987
${r}_{\mathrm{CO}}$	333–383	79.9	6.5 × 10^6^	0.973
	393–442	36.6	8.83	0.988

Oxygen consumption activation energy increase (39.7 to 51.1 kJ/mol): In the temperature range of 333–383 K, oxygen mainly undergoes physical adsorption and weak chemisorption on active sites of the coal surface, resulting in a low apparent activation energy (39.7 kJ/mol). As the temperature rises to 393–442 K, adsorption gradually becomes saturated, and the rate‑controlling step shifts to chemical bond cleavage requiring higher energy, such as the oxidation of aliphatic side chains and the decomposition of peroxides. Consequently, the apparent activation energy increases to 51.1 kJ/mol.

CO generation activation energy decrease (79.9 to 36.6 kJ/mol): In the 333–383 K range, CO is primarily produced by the thermal decomposition of aldehyde (–CHO) and carboxyl (–COOH) groups, a process that must overcome a relatively high bond energy (79.9 kJ/mol). At further elevated temperatures, the oxygen-containing intermediates formed on the coal surface (e.g., peroxy radicals) decompose more rapidly, and CO generation gradually becomes controlled by product desorption or diffusion through the coal pore structure. These physical processes exhibit low activation energies (approximately 30–40 kJ/mol), leading to a marked decrease in the apparent activation energy.

### Determination of heat release intensity

**Fig. 2:**
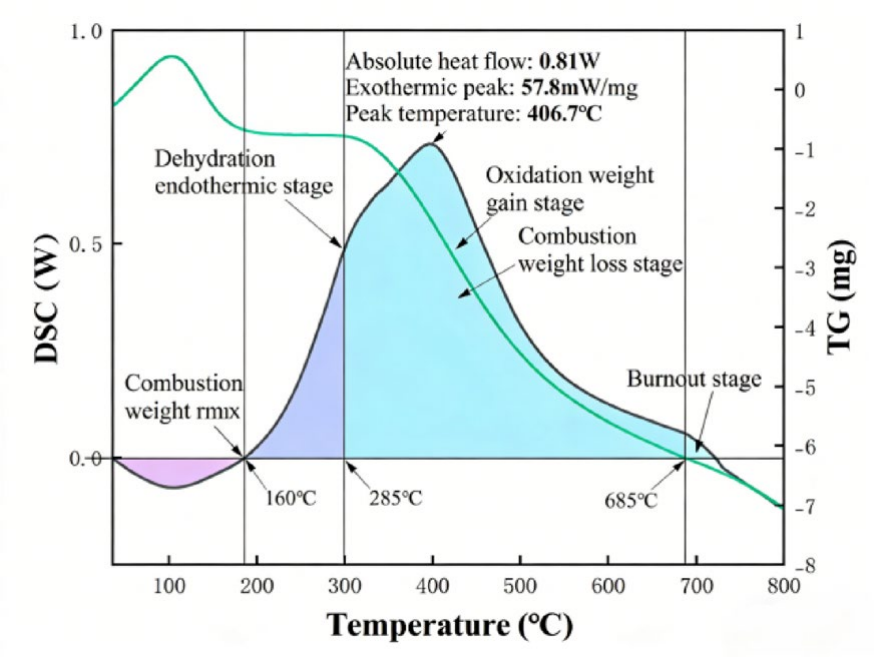
TG-DSC curves of coal spontaneous combustion.

The heat release intensity is an important indicator for evaluating the exothermic characteristics of coal. Its calculation and analysis are essential and also provide necessary data support for subsequent COMSOL simulations of the temperature rise caused by coal oxidation in goafs. In this study, a simultaneous thermal analyzer (STA) was used to perform synchronous thermal analysis experiments on the coal sample. Approximately 14 mg of coal with a particle size of 80–100 mesh was placed in the instrument and heated to 800 °C at a heating rate of 15 °C/min. The TG-DSC curves of the coal sample as a function of temperature were obtained, as shown in Fig. 2.

The coal sample underwent a dehydration and endothermic stage between 33 and 160 °C. In this temperature range, the DSC curve lies below the baseline, showing a distinct endothermic peak, while the TG curve decreases slowly, indicating that the moisture in the coal evaporates upon heating, resulting in mass loss and heat absorption.

In the range of 160–285℃, the coal sample enters the oxidation and mass gain stage. the TG curve showed a slight upward trend, and the DSC curve transitioned from endothermic to exothermic, suggesting that low-temperature oxidation reactions begin to dominate the thermal behavior.

The temperature range of 285–685 °C corresponds to the combustion and mass loss stage, during which the coal sample undergoes intense oxidative combustion. The TG curve drops sharply, and the DSC curve exhibits a significant and strong exothermic peak. The peak exothermic rate occurs at approximately 406.7℃, with a maximum value of 57.8 mW/mg. Based on the experimental results, the heat release intensity of the coal sample from the working face was calculated to be 78.03 J/(cm^3^ s).

Since the synchronous thermal analysis experiment exhibits an endothermic behavior in the low-temperature range due to the evaporation of moisture in the coal sample, the results cannot be directly used as the heat source input for numerical simulation. Therefore, the present study also employed the bond energy estimation method to derive a calculation formula for the heat release intensity, thereby obtaining a heat source term suitable for simulation^18^.

3 $$Q\left( T \right) = \Delta H_{{{\mathrm{O}}_{2} }} \Delta r + H$$

4 $$\Delta r = r_{{{\mathrm{O}}_{2} }} \left( T \right) - r_{{{\mathrm{CO}}}} \left( T \right) - r_{{{\mathrm{CO}}_{2} }} \left( T \right)$$

5 $$H = \Delta H_{{{\mathrm{CO}}}} r_{{{\mathrm{CO}}}} (T) + \Delta H_{{{\mathrm{CO}}_{2} }} r_{{{\mathrm{CO}}_{2} }} (T)$$

**Fig. 3:**
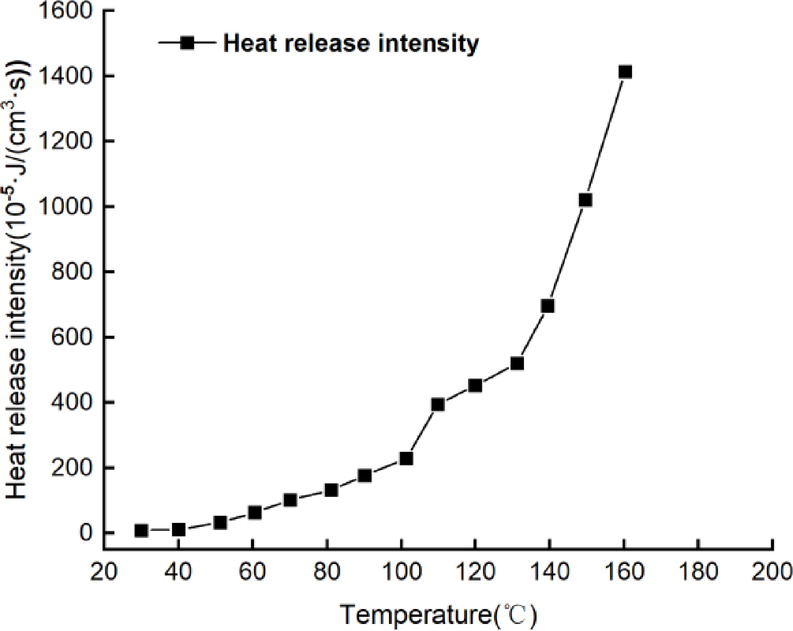
Heat release intensity as a function of temperature.

The heat release intensity of the coal sample from the working face was calculated using the aforementioned formula, and the relationship between heat release intensity and temperature is plotted in Fig. 3. It can be observed that the variation trend of the heat release intensity is almost identical to that of the oxygen consumption rate.

To quantitatively describe the temperature dependence of heat release intensity, the experimental data were fitted using a segmented Arrhenius model, and the corresponding kinetic parameters are summarized in Table 2.

**Table 2:** Segmented Arrhenius parameters for heat release intensity.

Segment	*T*	${E}_{a}$	*A*	${R}^{2}$
*Q(T)*	303–383	46.2	1.08 × 10^10^	0.992
	393–433	40.9	1.95 × 10^9^	0.998

## Simulation analysis of CO migration laws in goaf areas

### Establishment of mathematical models

The residual coal in the goaf is essentially a typical porous medium, and the gas flow within it must obey the fundamental physical laws of mass conservation, energy conservation, and momentum conservation^19^. Meanwhile, due to the mixing and diffusion of multiple gas components, the process must also satisfy the requirement of species conservation. Based on these conservation laws, a set of differential governing equations describing the migration and diffusion behavior of CO in the goaf can be derived. By further specifying the corresponding boundary conditions and initial conditions, these equations together form a mathematical model capable of systematically characterizing the gas flow characteristics in the goaf.

1. Flow field control equation:
  6 $$\frac{\mu }{k}{\textbf{u}} = - \nabla p + \mu \nabla^{2} {\textbf{u}} + \rho_{f} {\textbf{g}} + {\textbf{F}}$$
  7 $$\frac{{\partial \left( {n\rho_{f} } \right)}}{\partial t} + \nabla \cdot \left( {n\rho_{f} {\textbf{u}}} \right) = S_{{\mathrm{m}}}$$
2. Component transport equation:
  8 $$\frac{{\partial c_{i} }}{\partial t} + \nabla \cdot \left( { - D_{i} \nabla c_{i} } \right) + {\textbf{u}} \cdot \nabla c_{i} = R_{i}$$
3. Temperature field control equation:
  9 $$\left( {\rho c_{p} } \right)_{eff} \frac{\partial T}{{\partial t}} + \rho_{f} c_{p,f} u \cdot \nabla T + \nabla \cdot \left( { - k_{eff} \nabla T} \right) = Q$$
4. Ideal gas state equation:
  10 $$\rho = \frac{pM}{{RT}}$$

### Establishment of physical model and setting of boundary conditions

**Fig. 4:**
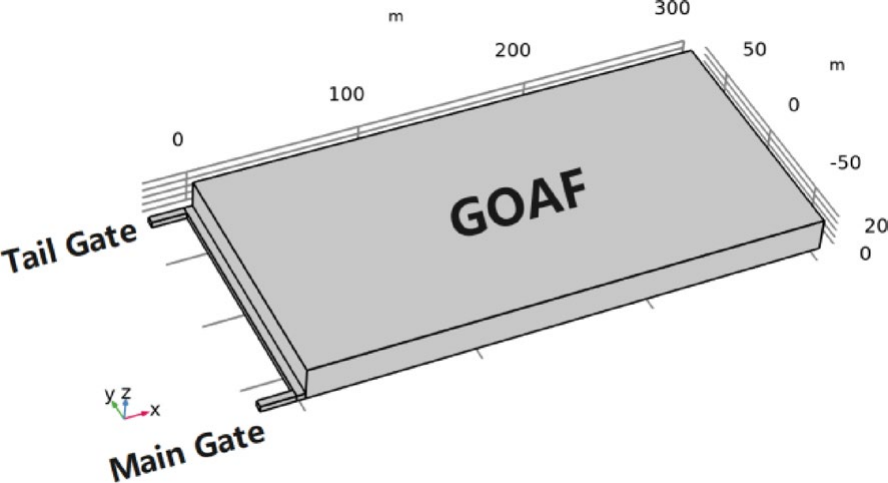
Physical goaf model for Working Face.

The U-shaped ventilation model used in this study is shown in Fig. 4. A dip length of 150 m, a vertical height of the goaf of 20 m, and an extent along the strike of 300 m. This model height is determined based on the classical “vertical three zones” theory of overburden movement, which fully covers the core region of coal oxidation and CO migration for the 2.5 m thick coal seam studied in this work. Both the intake and return airways in the model adopted a rectangular cross-section of 3.5 m × 4.5 m, with a uniform length of 20 m.

It should be noted that the goaf is a dynamically collapsed, heterogeneous and inaccessible fractured rock mass. Restricted by overburden movement characteristics and underground safety constraints, it is technically impossible to conduct full-scale in-situ measurement of porosity and permeability at different positions inside the goaf. Therefore, the combination of field statistical average values and classical empirical formulas is the universally accepted and widely adopted parameter determination method in the field of goaf numerical simulation worldwide.

Permeability directly affects the intensity of gas flow in the goaf, and its magnitude is closely related to the porosity of the goaf. As the working face advances, the overlying strata in the goaf continuously collapse, undergoing a transition from stability to instability and then back to stability. Owing to different collapse conditions in various regions, the porosity within the goaf exhibits an “O”-shaped pattern^20^. The bulking factor is defined as the rate of volume increase of loose rock relative to its original state. The relationship between porosity and the bulking factor is as follows:

11 $$n = \left( {1 - \frac{1}{{K_{{\mathrm{p}}} }}} \right)$$

12 $$K_{{\mathrm{p}}} = K_{{{\mathrm{p}},\max }} - \left( {K_{{{\mathrm{p}},\max }} - K_{{{\mathrm{p}},\min }} } \right) \cdot \Delta$$

13 $$\Delta = e^{{ - a_{1} d_{1} \left( {1 - e^{{ - c_{1} a_{0} d_{0} }} } \right)}}$$

The relationship between permeability and porosity conforms to the Blake-Kozeny formula^21^, Overburden zone permeability

14 $$k = \frac{{D_{p}^{2} n^{3} }}{{180(1 - n)^{2} }}$$

**Fig. 5:**
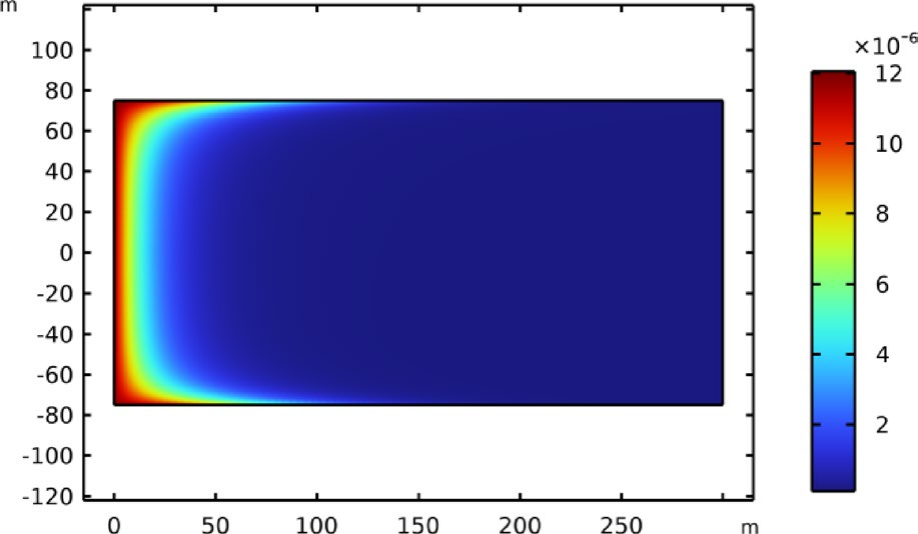
The permeability distribution in the goaf.

The permeability distribution in the goaf is shown in Fig. 5.

Based on the field measurements, the average wind velocity in the intake airway of the working face is set to 1.6 m/s. The residual coal thickness was set at 0.5 m in the two roadway areas and 0.2 m in the central area. Since the coal seam dip angle is only 16°, it has no significant influence on the thickness of residual coal in the goaf. The goaf is assigned an initial temperature of 30 °C. The oxygen consumption rate, CO generation rate and heat release intensity obtained from the programmed heating experiments are incorporated into user-defined functions to simulate the heat generation and CO production during coal oxidation in the goaf.

### Basic assumptions

Given the complexity and dynamic nature of the spontaneous combustion process in goaf areas, achieving convergence in simulations is often challenging and typically incurs substantial computational costs. To simplify the simulation procedure without significantly compromising the accuracy of the model results, the following assumptions are proposed:

1. Local thermal equilibrium is assumed for heat transfer in porous media;
2. gravity, thermal buoyancy, and coal seam dip angle effects are taken into account.

### Solver methods and mesh generation

In this study, the Free and Porous Media Flow, Diluted Species in Porous Media, and Heat Transfer in Porous Media modules are employed to solve the velocity field, gas concentration field, and temperature field, respectively. Owing to the inclusion of gravity, thermal buoyancy, and coal seam dip angle effects, the model became highly nonlinear. To address this issue, a non-isothermal flow multiphysics module is introduced to solve the temperature and flow fields. Subsequently, a segregated solver is employed to separately solve the non-isothermal flow and the concentration field.

**Fig. 6:**
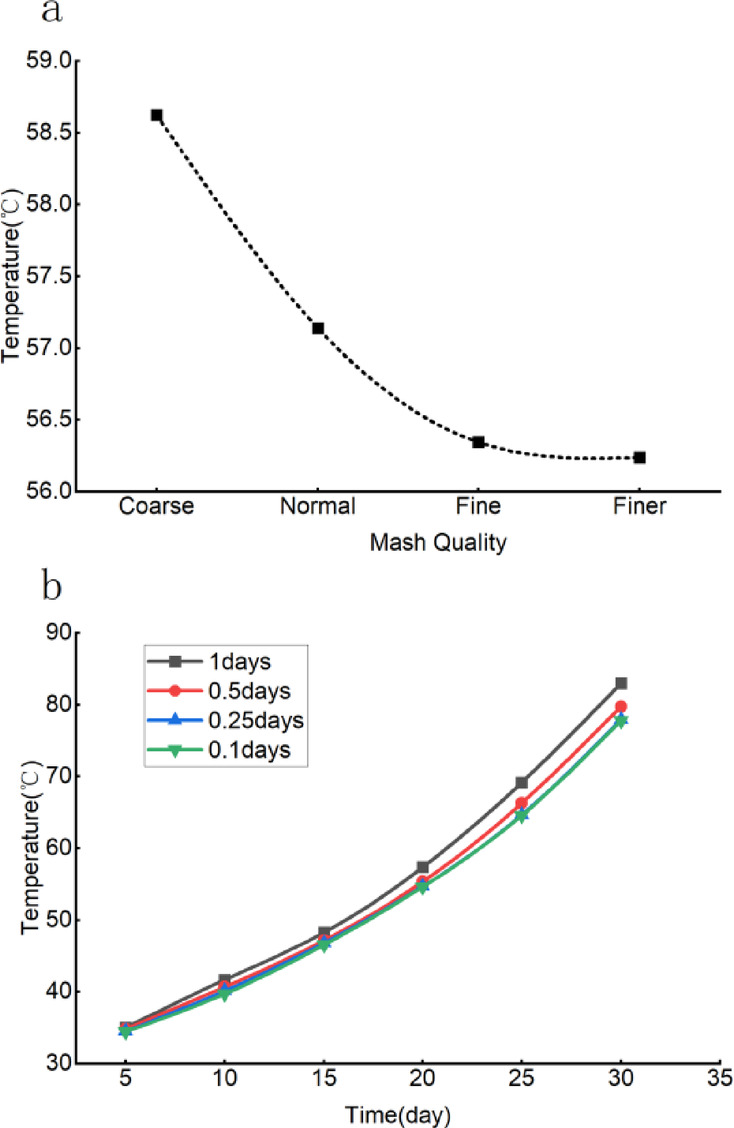
Sensitivity tests of mesh (**a**) and time step (**b**) in sponcom simulations.

To ensure model accuracy and numerical simulation efficiency, grid independence and time step sensitivity tests were conducted on the model, with the results presented in Fig. 6. In the multiphysics coupling, temperature is a key factor influencing indicators such as the CO generation rate and the oxygen consumption rate. Therefore, independence validation was performed for four different grid resolutions (coarse, normal, fine, and finer). As shown in Fig. 6a, when the grid resolution reached “fine”, the error between the results and those obtained with the extra-fine grid was less than 5%, and further refinement had a negligible effect on the results^22^. Based on this optimal grid resolution, the time step independence was verified using time steps of 1 day, 0.5 days, 0.25 days, and 0.1 days. As can be seen from Fig. 6b, when the time step was 0.25 days, the error relative to the 0.1 days time step was less than 5%. Accordingly, a time step of 0.25 days was selected for this study.

### Model validity verification

Oxygen concentration is the most fundamental and direct indicator reflecting the intensity of coal oxidation and oxygen consumption in the goaf. The division of the spontaneous combustion three zones based on oxygen concentration is a widely recognized, classical and reliable method in the field of coal mine fire prevention and control. Since oxygen migration and consumption are the core physical and chemical processes governed by the numerical model, the oxygen concentration distribution is the most sensitive to model parameters such as air leakage intensity, porosity and oxygen consumption rate. Therefore, comparing the three-zone ranges obtained from numerical simulation and field monitoring can directly reflect whether the model truly reproduces the actual oxygen migration law and oxidation environment in the goaf. As shown in Table 3, the distribution characteristics of the three zones from field measurements and numerical simulation were basically consistent, which verifies that the numerical model is reliable.

**Table 3:** Comparison of the spontaneous combustion three zones between field monitoring and numerical simulation.

Division of three zones	Criterion	Field monitoring data	Numerical model
Heat dissipation zone	O_2_ > 7.48 mol/m^3^ (18%)	Intake side: 0–30 m	Intake side: 0–35 m
Oxidation zone	O_2_ between 7.48 mol/m^3^ (18%) and 3.32 mol/m^3^ (8%)	Intake side:30–140 m	Intake side:35–150 m
		Return side:20–60 m	Return side:20–60 m
Inert zone	O_2_ < 3.32 mol/m^3^ (8%)	Intake side: > 140 m	Intake side: > 150 m
		Return side: > 60 m	Return side: > 60 m

As an important oxidation product of residual coal, CO generation, accumulation and migration are essentially controlled by the oxygen transport, temperature variation and airflow migration within the goaf, which are fully embodied by the spatial scope of the spontaneous combustion three zones. The oxidation zone determined by oxygen concentration is exactly the main occurrence region of CO generation, while the heat dissipation zone and inert zone dominate the dispersion and accumulation characteristics of CO gas. Restricted by the complex geological conditions and broken overburden of the goaf, it is impractical to arrange precise and undisturbed internal monitoring points to obtain reliable in-situ CO concentration data. Meanwhile, CO measurements from roadways and shallow boreholes are easily interfered by underground ventilation fluctuation and external gas sources, which cannot objectively represent the original CO migration pattern inside the goaf. Consequently, the good consistency of the three-zone division can indirectly and reasonably validate the rationality of the simulated CO generation and migration characteristics, and this verification strategy is widely adopted in similar goaf multi-physics simulation studies.

## Analysis of numerical simulation results

### Distributions of temperature and oxygen concentration in the goaf

**Fig. 7:**
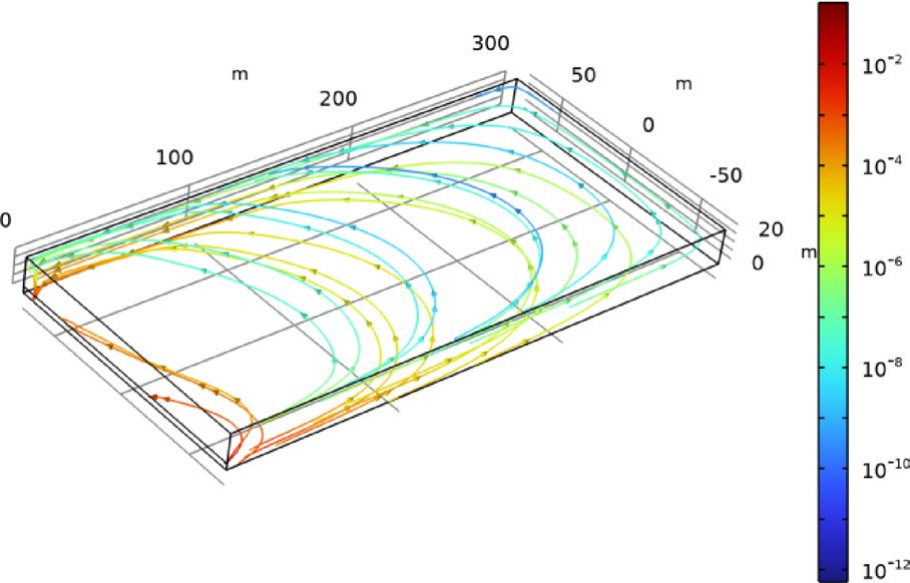
Velocity streamline distribution in the goaf.

Figure 7 shows the flow field distribution in the goaf. The velocity of the air leakage flow in the goaf is a key factor influencing spontaneous combustion of residual coal. It directly determines the spatial distributions of oxygen supply, heat accumulation, and the oxidation reaction zone. Even minor variations in air velocity can lead to significant fluctuations in the risk of spontaneous combustion in the goaf. As can be seen from the figure, air leakage in the goaf mainly occurs on the intake side, which is also the region with the highest flow velocity in the entire goaf. After entering the interior of the goaf, the airflow velocity decays exponentially and gradually assumes a symmetric distribution. The effect of flow velocity on spontaneous combustion is a nonlinear process, in which both excessively low and excessively high velocities are unfavorable for spontaneous combustion.

**Fig. 8:**
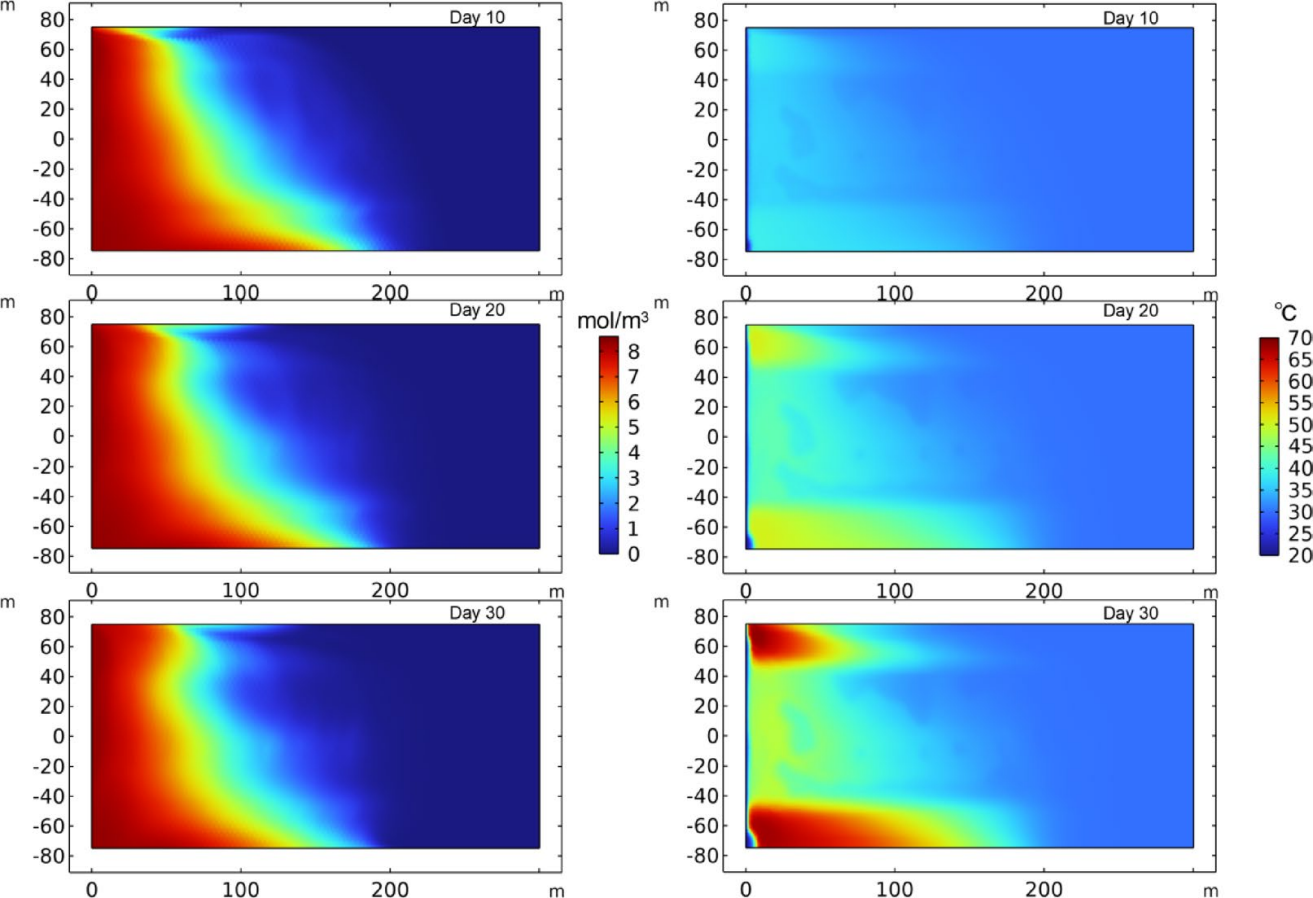
Oxygen concentration (left) and Temperature (right) contours after different reaction times.

Figure 8 shows the distributions of the temperature field and oxygen concentration field at the floor of the goaf at different time periods. The temperature in the goaf exhibits a trend of being higher on both sides and lower in the middle, with an obvious tailing phenomenon in the high-temperature zone. This phenomenon is influenced by two factors. On one hand, the porosity on both sides is greater than that in the middle, resulting in a higher oxygen concentration. On the other hand, it is also related to the thickness distribution of the residual coal; the residual coal thickness in the middle was only 0.2 m, so its capacity for heat generation and heat accumulation is significantly lower than that on both sides.

The oxygen concentration field is affected by both airflow and temperature. As the temperature increases, oxygen consumption gradually rises, and oxygen is rapidly consumed in the shallow goaf. Consequently, the oxidation zone moves toward the front (shallow part) of the goaf, while the inert zone expands continuously toward the deep goaf. On the return side, the thermal buoyancy gradually increases and drives the gas flow toward the return side. In the later simulation stage (after 20 days), the thermal buoyancy effect becomes more significant, and the oxidation zone near the return side further shrinks toward the shallow area.

### Analysis of CO migration patterns in the goaf

**Fig. 9:**
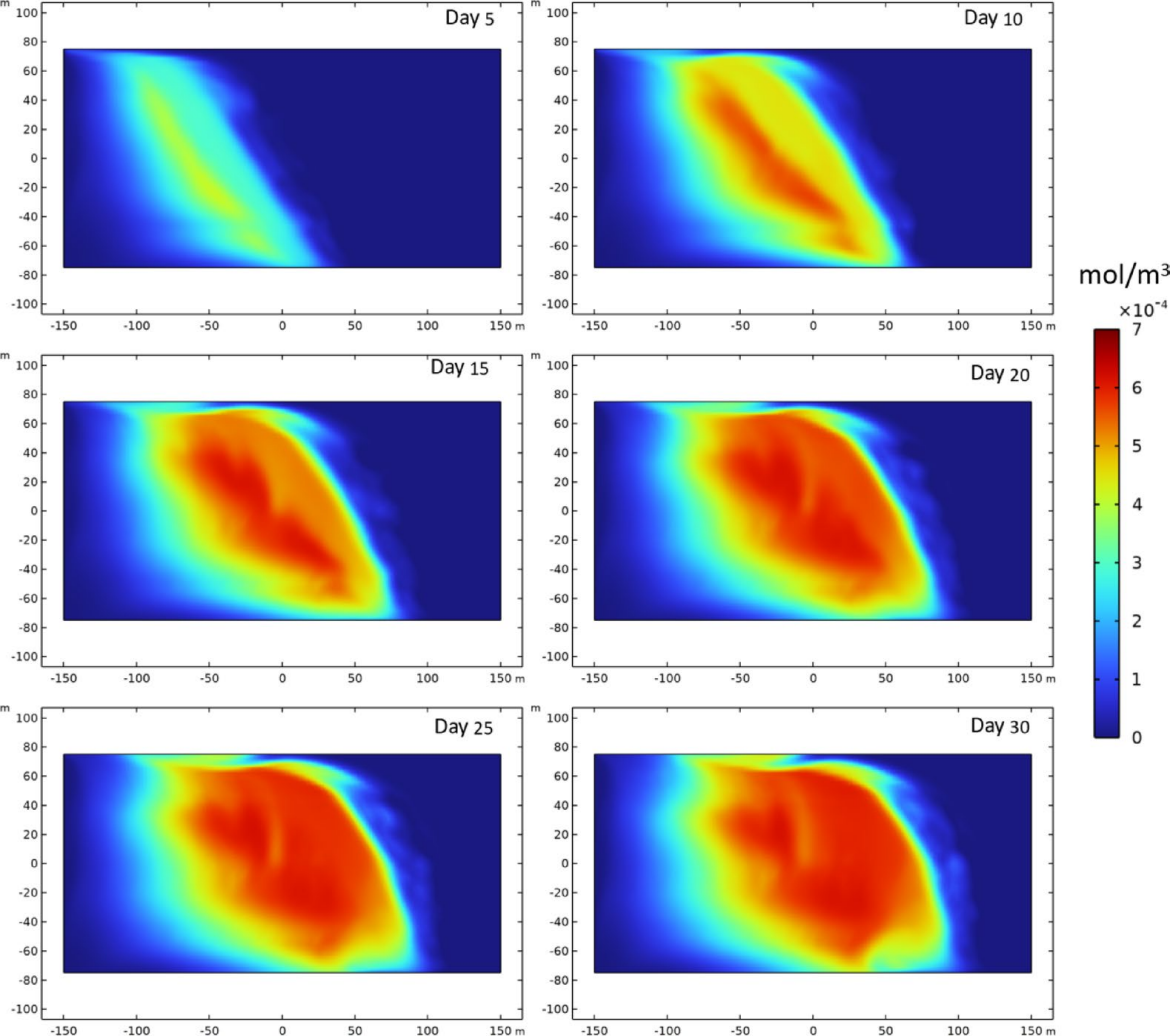
Spatiotemporal evolution of the CO concentration field in the goaf (plane Z = 0.1).

Figure 9 illustrates the migration patterns of the CO concentration field at the floor from Day 5 to Day 30. From a spatial perspective: In the heat dissipation zone, the high air leakage velocity enables the generated CO to be rapidly carried out of the goaf through the return side, resulting in an extremely low CO concentration in this area. In the oxidation zone, the airflow velocity decreases significantly, making it difficult for the CO generated by residual coal oxidation to be completely discharged. Instead, part of the CO is transported into the deep goaf and accumulates, with the peak CO concentration occurring from the middle of the oxidation zone to the interface between the oxidation zone and the inert zone. In the inert zone (suffocation zone), the airflow velocity further decreases and nearly stagnates. Due to the extremely low oxygen concentration in this zone, the oxidation reaction of residual coal is basically terminated, and no new CO is generated. All CO present here is transported from the oxidation zone via the flow field.

**Fig. 10:**
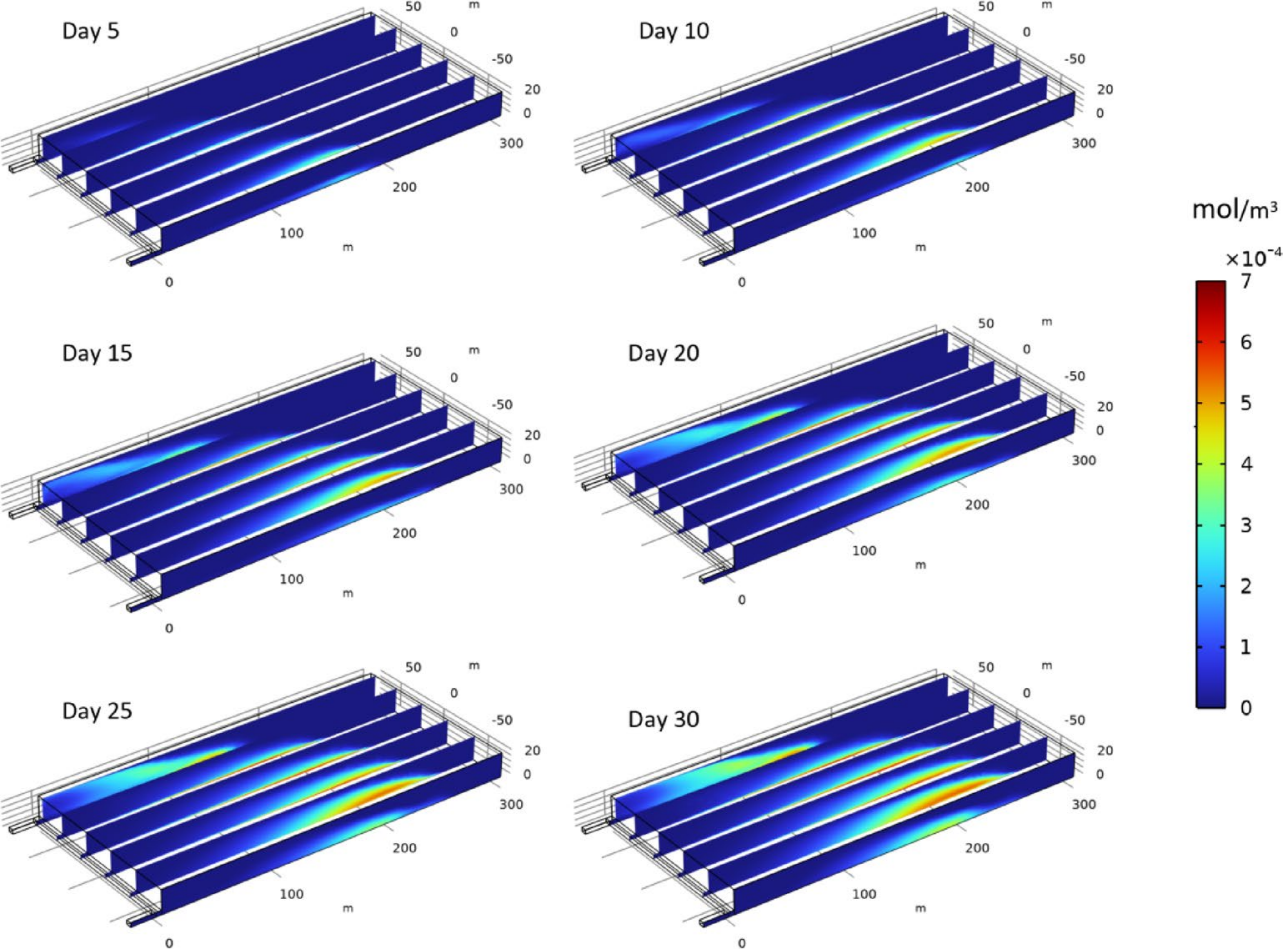
Spatiotemporal evolution of the CO concentration field in the goaf (plane Y = − 75, − 45, − 15, 15, 45, 75).

From a temporal perspective, during the first 15 days, the flow field was primarily driven by the pressure difference between the intake and return sides. After 15 days, as the temperature rises and the oxidation reaction rate accelerates, oxygen consumption increases sharply, causing the oxidation zone to move toward the front (shallow part) of the goaf, while the range of the inert zone expands. Concurrently, the effect of thermal buoyancy gradually intensifies, particularly on the return side. CO accumulated in the deep goaf is driven by thermal buoyancy toward the return side, which is consistent with the vertical migration law shown in Fig. 10.

Figure 10 shows the spatiotemporal evolution of the CO concentration field in the goaf at different vertical planes (Y =  − 75, − 45, − 15, 15, 45, 75). Based on the CO migration characteristics on the goaf floor (Fig. 10), this figure further reveals the evolution law of CO concentration along the height direction under the combined effects of gravity and thermal buoyancy.

To quantitatively evaluate the dominant role of thermal buoyancy in CO vertical migration, the Rayleigh number (*Ra*) for porous media and the buoyancy-to-forced-flow ratio (B/F) were calculated. The Rayleigh number characterizes the relative strength of natural convection driven by thermal buoyancy versus viscous dissipation, and its critical value for the onset of natural convection in porous media is approximately 1000. The buoyancy-to-forced-flow ratio represents the ratio of buoyancy driving force to forced convection driving force caused by ventilation pressure difference.

The Rayleigh number is defined as:

15 $$Ra = \frac{g\beta \Delta TKH}{{\nu \alpha }}$$

The buoyancy-to-forced-flow ratio is defined as:

16 $$\frac{B}{F} = \frac{\rho g\beta \Delta TH}{{\mu u/L}}$$

Under the simulation conditions of this study, the calculated average Rayleigh number was approximately 810, which is close to the critical value for natural convection. It should be noted that the temperature difference in the central oxidation zone can reach up to 40 °C, and the local Rayleigh number in this high-temperature region exceeds 1000, indicating that natural convection has been initiated. Meanwhile, the buoyancy-to-forced-flow ratio is calculated to be about 2.7, indicating that thermal buoyancy exceeded the forced convection driven by ventilation pressure difference and become the dominant factor controlling the vertical migration of CO.

From the perspective of vertical distribution, the CO concentration in the goaf shows a significant stratification and upward expansion phenomenon with height. In the early stage of the simulation (before Day 10), the CO is mainly concentrated near the goaf floor, with a low concentration and narrow distribution range in the upper part, and the migration is dominated by the pressure difference between the intake and return sides. As the temperature rises (after Day 15), the thermal buoyancy effect gradually becomes the dominant driving force for CO migration. The low-density CO is continuously driven upward, and the high-concentration CO zone gradually expands from the floor to the upper part of the goaf, with the vertical distribution range continuously increasing. In the later stage of the simulation (after Day 20), the distribution range of the high-concentration CO zone along the height direction expands significantly, extending continuously from the floor to the upper part of the goaf and forming a large-scale high-concentration enrichment zone at the top of the return side.

## Conclusion

Based on the numerical simulation of coal spontaneous combustion in the goaf, this paper systematically analyzes the spatiotemporal evolution characteristics of the flow field, temperature field, and CO concentration field within the goaf. Combined with quantitative hydrodynamic calculation and mining vertical three-zone theory, the core coupling mechanisms governing CO migration and distribution are fully revealed. The main conclusions are summarized as follows:

1. According to programmed heating experimental results, CO is almost exclusively generated by low-temperature coal oxidation with negligible desorption background in the study coal seam, which can be reliably adopted as the optimal indicator gas for monitoring coal spontaneous combustion inside the goaf.
2. The horizontal migration of CO is mainly dominated by the ventilation pressure gradient and air leakage flow. For the vertical migration behavior, quantitative calculation results show that the average Rayleigh number of the goaf is 810 (local value exceeding 1000 in the high-temperature oxidation zone), and the buoyancy-to-forced-flow ratio reaches 2.7 under the maximum temperature difference of 40 °C (30 °C to 70 °C). Thermal buoyancy is verified as the dominant driving force for vertical CO migration, which fundamentally controls the vertical expansion range and spatial concentration distribution of CO in the goaf.
3. Under the coupled effect of horizontal air leakage forced convection and vertical thermal buoyancy-driven natural convection, CO generated by residual coal oxidation gradually accumulates in the upper zone, deep region, and return airway side of the goaf. These areas are determined as the key monitoring zones for real-time CO concentration early warning and spontaneous combustion prevention.

It should be noted that this study still has certain limitations. The numerical simulation was carried out under idealized geological and mining conditions, without considering the dynamic variation characteristics of goaf porosity and residual coal particle size during continuous mining. In addition, the coupling mechanism of thermal buoyancy and air leakage under extreme working conditions needs further in-depth exploration. Future research will optimize the numerical model to be more consistent with actual on-site conditions and further enrich the multi-factor synergistic regulation mechanism of CO migration.

## Data Availability

The data that support the findings of this study are available from the corresponding author upon reasonable request.

## List of symbols

*A*: Pre-exponential factor (S^−^^1^)
C: Concentration of gas (mol/m^3^)
${c}_{p,f}$: Specific heat capacity of fluid at constant pressure (J/(kg K))
D: Diffusion coefficient (m^2^/s)
*Dp*: Particle diameter of porous medium (m)
*Ea*: Activation energy (kJ/mol)
F: Volume force (N/m^3^)
g: Acceleration of gravity (m/s^2^)
*H*: Intermediate heat term (W/m^3^)
*ΔH*: Average reaction heat (kJ/mol)
*k*: Permeability (m^2^)
*Kp*: Bulking factor
*n*: Porosity
*p*: Pressure (Pa)
${Q}_{heat}$: Energy source term (heat) (W/m^3^)
${Q}_{air}$: Air supply flow rate (mL/min)
Q: Heat release intensity (J/(cm^3^ s))
*R*: Ideal gas constant (J/(mol K))
*r*: Reaction source of gas (mol/(m^3^ s))
*Sm*: Mass source term (kg/(m^3^ s))
*T*: Thermodynamic temperature (K)
$\textbf{u}$: Fluid velocity vector (m/s)
*μ*: Fluid dynamic viscosity (Pa s)
*ρ*: Gas density (Kg/m^3^)
$\beta^{-1}$: Thermal expansion coefficient of air (K^−1^)
